# Skin temperature changes in wild chimpanzees upon hearing vocalizations of conspecifics

**DOI:** 10.1098/rsos.160816

**Published:** 2017-01-25

**Authors:** Guillaume Dezecache, Klaus Zuberbühler, Marina Davila-Ross, Christoph D. Dahl

**Affiliations:** 1 Institute of Biology, University of Neuchâtel, Neuchâtel, Switzerland; 2 Budongo Conservation Field Station, Masindi, Uganda; 3 Psychology Department, University of Portsmouth, Portsmouth, UK; 4 School of Psychology and Neuroscience, University of St Andrews, St Andrews, UK

**Keywords:** infrared thermography, skin temperature, wild chimpanzees, vocalizations, emotions

## Abstract

A growing trend of research using infrared thermography (IRT) has shown that changes in skin temperature, associated with activity of the autonomic nervous system, can be reliably detected in human and non-human animals. A contact-free method, IRT provides the opportunity to uncover emotional states in free-ranging animals during social interactions. Here, we measured nose and ear temperatures of wild chimpanzees of Budongo Forest, Uganda, when exposed to naturally occurring vocalizations of conspecifics. We found a significant temperature decrease over the nose after exposure to conspecifics' vocalizations, whereas we found a corresponding increase for ear temperature. Our study suggests that IRT can be used in wild animals to quantify changes in emotional states in response to the diversity of vocalizations, their functional significance and acoustical characteristics. We hope that it will contribute to more research on physiological changes associated with social interactions in wild animals.

## 1. Background

The question of whether emotional states can be attributed to non-human animals has a long history in science [1]. Traditionally, the description of animal behaviour in terms of emotional states has been discouraged on the grounds that such constructs were circular and fictional explanations of animal behaviour with no heuristic value [2,3]. For other researchers, however, behaviour in both human and non-human animals is driven by underlying psychological states, including emotions, which are accessible to systematic empirical investigation [4–7]. In fact, it is legitimate to attribute emotions to non-human organisms once they are defined as the neural responses to specific stimuli and their associated and observable motoric and physiological outputs, without requiring that animals possess a subjective perception of these bodily changes [8]. If emotions are a valuable concept to describe animal behaviour, then their physiological correlates must be reliably measured and evaluated in non-verbal animals, independently of the social behaviour they are allegedly driving. A similar line of argument has been used to introduce other psychological concepts, such as friendship [9], indicating that using notions from human social experience to describe animal behaviour has become increasingly acceptable [6].

The main empirical approach to study emotions in animals is to monitor the activity of the autonomic nervous system as a proxy of emotional states, using cardiovascular, electro-dermal and respiratory responses (see [10] for a review). This line of research has produced evidence of physiological reactions to a range of social stimulations [11–13], but only under highly controlled laboratory conditions. In addition, such measurements impose drastic limitations, for instance, by restricting the movements of subjects and requiring tolerance to electrodes, which rules out studies in free-ranging animals in ecologically valid contexts (but see [14]).

The development of infrared thermography (IRT) has brought the possibility to break this constraint. IRT is a contact-free method, which calculates the temperature of an object from the infrared radiation received within the imager's field of view [15]. Changes in body surface temperature reflect shifts in blood flow to the surface of the skin, a process that is controlled by the autonomic nervous system. This process is mediated by cutaneous vasoconstriction, which reduces the blood flow to certain body areas and results in those body parts cooling down [16].

In humans, various studies have documented changes in facial skin temperature as responses to affective situations, such as sexual arousal to tactile stimulations [17], inflictions of guilt [18], or empathic responses to distress in mother–infant dyads [19]). Regions of interest (ROIs) in humans usually are the forehead as well as the nasal, perinasal, orbital, periorbital and maxillary regions of the face (see [20] for a recent and comprehensive review). Those studies, for instance, report a drop in nose and maxillary area temperatures under stressful and fearful stimulations [20]. In particular, the tip of the nose is thought to offer reliable insights into the cutaneous and subcutaneous variations in blood flow, owing to its rich blood vessel supply.

In non-human animals, skin temperature changes following emotional stimulations have also long been reported [21]. In one study, facial imprints of captive rhesus monkeys were monitored, as they were approached by a threatening individual (a veterinarian). A drop in nasal temperature was observed within seconds after stimulation [22]. In a subsequent study, Kuraoka & Nakamura [23] found similar effects on nasal temperature within 20 s after the onset of a video depicting an aggressive conspecific. More recently, Kano *et al*. [24] found that captive chimpanzees, exposed to sounds and videos of agonistic interactions involving conspecifics, also showed a drop in nasal temperature. This shift in temperature was related to heart-rate responses and was thus supposed to reflect the activity of the sympathetic nervous system [24]. Importantly, Kano *et al*. controlled for movement artefacts thus providing evidence that shifts in nasal temperature indicated changes in emotional states rather than locomotor responses. However, the nasal part of the face may be problematic for assessing underlying psychological activity, for two reasons. First, in primates and a number of other species, direct and prolonged gaze is a signal of aggressive intent, so experimenters are often barred from directly facing the subject, a prerequisite of IRT. Second, the nasal temperature is largely affected by the breathing pattern, with increased breathing causing significant and rapid cooling of the nose, a pattern of activity which might erratically be associated with shifts in the emotional state of the animal. However, a recent study [25] observed rising temperature in the nasal region in situations (such as crying) where increased breathing is typically expected.

In our study, the aim was to assess emotional states of chimpanzees upon hearing vocalizations of conspecifics. Although IRT has already been performed in wild animals [26,27], we are not aware of any study that has investigated responses to natural stimuli in free-ranging animals. We looked at chimpanzees' response to a range of naturally occurring vocalizations. We hypothesized that vocalizations associated with aversive contexts (such as screams, whimpers and aggressive barks) may yield to more consistent changes in skin temperature than vocalizations used in peaceful and relaxed contexts (such as travel and resting hoos) [28], as they are associated with urgent social challenges that require fast responses. Additionally, we were able to obtain IRT readings following vocalizations from a neighbouring community, which represents a most threatening situation in wild chimpanzees [29]. We hypothesized that temperature shifts when exposed to calls from a neighbouring community were more consistent compared with calls from the subject's own community given the differences in arousal they are associated with. To address these hypotheses, we consistently monitored the skin temperature of the ears and nasal region. Our general assumption was that shifts in ear temperature were concomitant with shifts in nose tip temperature, owing to the same underlying processes in the autonomic nervous system, i.e. changes in blood flow at the surface of the skin in response to local muscular demands.

## 2. Material and methods

### 2.1. Study site

The study was carried out in the Budongo Forest Reserve, a moist, semi-deciduous tropical forest in Western Uganda, covering 428 km^2^ at an altitude of 1100 m, between 1°35′ and 1°55′ N and 31°08′ and 31°42′ E [30]. Data were collected on the Sonso chimpanzee community between February and June 2014, in December 2014 and between April and May 2016. Habituation of this community to humans started in 1990, with the majority of about 70 individuals well habituated to human observers [31]. The Sonso chimpanzees are Eastern chimpanzees (*Pan troglodytes schweinfurthii*), a subspecies of the common chimpanzee. Eastern chimpanzees live in multi-male multi-female societies marked by a fission–fusion dynamics (i.e. they travel and forage in small groups and reunite during the day). Males form long-lasting bonds and compete for hierarchy status and access to oestrous females. Females are less gregarious than males [32] and they usually leave the community once they reach sexual maturity [28].

### 2.2. Materials

Temperature measurements were collected with a Testo (881-2) thermal imager, which operates between 8 and 14 µm with a thermal sensitivity of less than 80 mK at 30°C. Emissivity was set at 0.98 [33]. A telephoto lens was used for all images (9° × 7°/0.5 m with a resolution of 1.0 mrad).

### 2.3. Data collection

**Figure 1.**
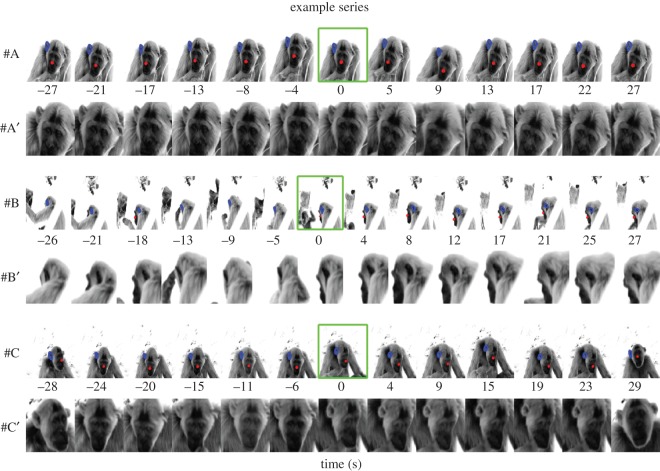
Example series of thermographic photographs. Horizontally aligned, series of pictures (with and without the ROI drawn) are presented as a function of time (*x*-axis). The ‘nose’ area is illustrated by a red outline, the ‘ear’ area by a blue outline. The green frame indicates the occurrence of the vocalization.

Data collection took place between 7.00 and 16.30 local time. We followed the main party (the largest group of individuals travelling and foraging together within a 30 m radius) in which most individuals were sufficiently tolerant to observer presence within 10 m, and collected thermographic pictures ad libitum. To be considered as a subject, animals had to be not under direct sunlight, move less than 10 consecutive metres for at least 1 min and not been exposed to vocalizations from conspecifics for at least 1 min. Once these conditions were met, the facial area of subjects was systematically photographed from a distance of less than 15 m. We took as many photographs as possible, with the aim of collecting at least one picture every 5 s until a vocalization was produced by a conspecific (figure 1). Note that we did not consider sounds that were clearly vegetative (such as breathing). After a vocalization was produced, we took as many pictures as possible for up to 30 s after the onset of vocalization, or until the subject moved away. If the subject moved more than 10 consecutive metres, the data collection was stopped. In the event that the subject was making a shift in posture (making the region of interest unavailable), we waited until they took their initial posture. We noted down the reference of the last picture before the vocalization was heard. We distinguished between six types of vocalizations as follows: screams/whimpers (*n* = 15), pant hoots (*n* = 23), aggressive barks (*n* = 4), travel and resting hoos (*n* = 14), grunts (*n* = 3) and pant-grunts (*n* = 19), see [28]. The call types are widely used categories within the chimpanzee vocal repertoire, identified by an experienced field assistant naive to the hypotheses. Any subsequent vocal behaviour of the subject (vocal response within 5 s after onset) was also noted down. Whenever possible, the identity of the caller as well as its distance from the subject were reported. In total, we were able to collect 78 such series of thermal pictures from 14 individuals (see table 1 for details).

**Table 1.** List of participants, number of series contributed, sex, year of birth (±*x* years).

identity	no. series contributed	sex	year of birth
FK	5	male	1999 ± 1 yr
HW	4	male	1993 ± 1 yr
KT	4	male	1993
KZ	5	male	1995
MS	5	male	1991
NB	6	female	1962 (estimation)
NK	3	male	1982 ± 1 yr
NT	5	female	2003
PS	8	male	1998 ± 1 yr
RS	3	female	1997
SQ	3	male	1991 ± 1 yr
ZF	7	male	1982
ZG	12	male	1997
ZL	8	male	1995
total	78		

### 2.4. Regions of interest

From each thermal image we extracted the nose and/or ear (see figure 1 for example series). We used the Matlab (Mathworks Inc.) ‘imfreehand’ function to draw a drag-able freehand region interactively using a computer mouse. We then extracted temperature values within the ROIs and determined the average values. Certain thermal images were filtered out if ROIs could not be reliably drawn (owing to, for instance, temporary shift in body orientation of the subject).

### 2.5. Data processing and analysis

**Figure 2.**
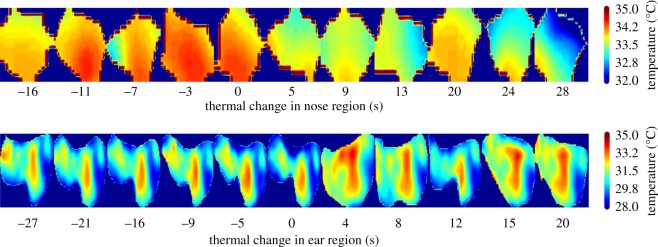
Time series of thermographic images with focus on nose and ear regions. From left to right two series of thermal images are shown as a function of time. The colour-code corresponds to the temperature values as indicated by the colour bar.

We subtracted the mean of all ROI temperatures of the series from the temperature values of each individual ROI value (e.g. figures 1 and 2). This procedure normalizes the data by using the mean as a reference level (baseline), while maintaining the original units of measurement (°C). Such a procedure was necessary to enable comparison between series, since a considerable number of factors (ambient temperature, humidity, distance-to-the-subject among others) may have impacted the amplitude of absolute temperature values (these basic measurements should be taken into account for future research on wild animals directly comparing between independent data points). After normalization, we temporally aligned values according to the relative time of shooting the thermal images and the vocalization occurring. We further pooled all values from thermal images occurring ‘before’ and ‘after’ the vocalization and calculated their mean for each subject. From all thermal images, we selected the ones that were inside a time period starting at −30 s before and ending 30 s after the vocalization. To illustrate the data distribution, we used a two-dimensional Gaussian smoothing kernel with a standard deviation of 2. Furthermore, we divided up all values from thermal images into two larger vocal categories: aversive (i.e. screams, whimpers, barks and pant hoots from the outgroup (nose, *n* = 24; ear, *n* = 40)) and non-aversive (i.e. resting and travel hoos (nose, *n* = 6; ear, *n* = 14)).

We applied two-tailed two-sampled *t*-tests under the null-hypothesis that the samples (before versus after) were drawn from the same distribution. We also applied Pearson linear correlation between the factors *time* and *temperature* using individual data samples (as opposed to the averaged values described above), i.e. all events in all series. We used these tests for the whole dataset as well as for parts of the dataset, such as for a specific vocalization type. We further applied Pearson linear correlation testing. Finally, we ran a bootstrapping procedure 1000 times, randomly sampling 10% of the data samples from the periods before and after the vocalization to verify the absence of biases and prediction errors.

## 3. Results

### 3.1. General pattern: before versus after a vocalization is produced

**Figure 3.**
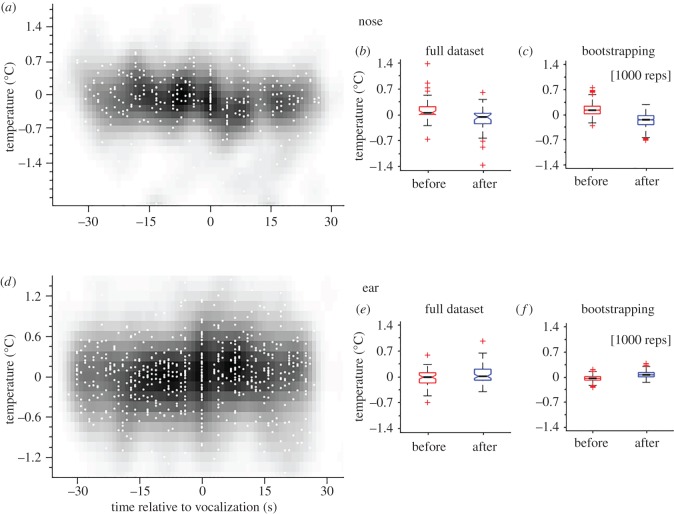
Full dataset, distribution and bootstrapping. (*a*,*d*) The full dataset is displayed in degree change from the baseline (*y*-axis) as a function of time (*x*-axis). Grey-scale clouds indicate the density of samples at given *x*- and *y*-values determined by Gaussian blurring. (*b*,*e*) The full dataset is shown as sample before (red) and after (blue) the occurrence of the vocalization. The black central line indicates the median, the box the first and third quartiles, whiskers the minimal and maximal values and asterisks the potential outliers. (*c*,*f*) Bootstrapped data are shown as the distribution of 1000 randomly chosen sets containing of 10% of the full dataset. Panels (*a*–*c*) display the changes of temperature in the nose, (*d*–*f*) display the changes of temperature in the ear.

We found that the temperature of the nose was significantly lower after than before the vocalization (figure 3*a*,*b*: *t*_39_ = 3.52, *p* < 0.001; mean: 0.13 ± 0.34 s.d. (before), −0.16 ± 0.39 s.d. (after)). By contrast, and contrary to our assumption, the temperature in the ear region significantly *increased* after a vocalization as opposed to before (figure 3*d*,*e*: *t*_71_ = −2.38, *p* < 0.05; mean: −0.04 ± 0.22 s.d. (before), 0.07 ± 0.25 s.d. (after)). To test our dataset for biases and prediction errors, we used a random sampling method (see Material and methods) and compared outputs of samples before and after the vocalization. We found a similar trend as for the full dataset, showing a decrease in temperature for the nose after the vocalization occurred (figure 3*f*: *t*_999_ = 39.28, *p* < 0.001; mean: 0.14 ± 0.16 s.d. (before), −0.16 ± 0.18 s.d. (after)) and an increase of temperature for the ear after the vocalization occurred (figure 3*f*: *t*_999_ = −30.23, *p* < 0.001; mean: −0.04 ± 0.07 s.d. (before), −0.06 ± 0.09 s.d. (after)).

### 3.2. Aversive versus neutral vocalizations

**Figure 4.**
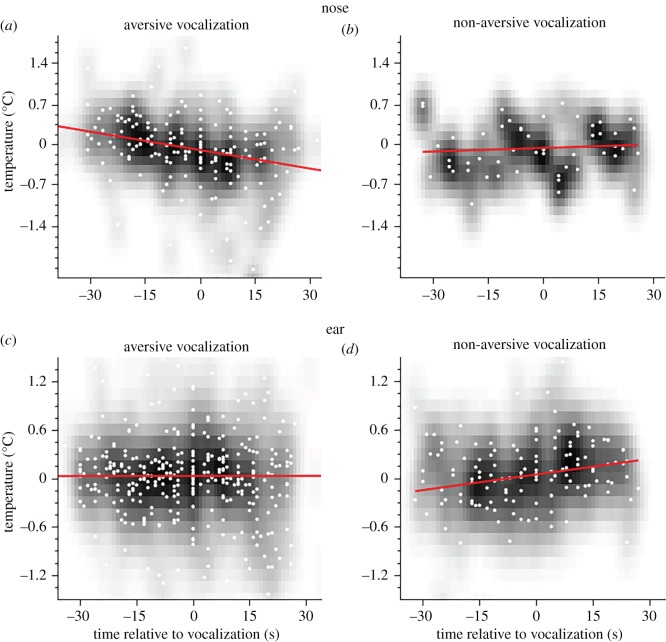
Aversive and non-aversive vocalizations. (*a*,*c*) Changes of temperature are displayed before and after the occurrence of an aversive vocalization. (*b*,*d*) Changes of temperature are displayed before and after the occurrence of a non-aversive vocalization. Colour-codes follow figure 3. Red lines show the Pearson linear correlation between changes in temperature and time.

In a subsequent analysis, we split the dataset according to the type of vocalization (aversive: ‘barks’, ‘screams’, ‘whimpers’ and ‘pant hoots’ of outgroup individuals versus non-aversive: ‘resting hoos’ and ‘travel hoos’). When comparing the period before and after a vocalization, aversive calls were associated with a large decrease in temperature of the nose (figure 4*a*: *t*_23_ = 2.85, *p* < 0.001; mean: 0.16 ± 0.40 s.d. (before), −0.23 ± 0.45 s.d. (after)), while non-aversive calls did not (figure 4*b*: *t*_5_ = 1.13, *p* = 0.31; mean: −0.15 ± 0.35 s.d. (before), 0.17 ± 0.36 s.d. (after)). The same patterns are visible when applying Pearson linear correlations between the two variables *time* and *temperature* (aversive: *r* = −0.29, *n* = 171, *p* < 0.001; non-aversive: *r* = 0.08, *n* = 50, *p* = 0.28).

In the ear region, we found no significant temperature change owing to aversive vocalizations (figure 4*c*: *t*_39_ = −0.47, *p* = 0.64; mean: 0.01 ± 0.21 s.d. (before), 0.03 ± 0.23 s.d. (after)), but a significant increase in temperatures after non-aversive vocalizations (figure 4*d*: *t*_13_ = −2.29, *p* < 0.05; mean: −0.09 ± 0.19 s.d. (before), 0.10 ± 0.25 s.d. (after)). The same pattern was found in correlation coefficients between *time* and *temperature* (aversive: *r* = 0.01, *n* = 377, *p* = 0.49; non-aversive: *r* = 0.20, *n* = 134, *p* < 0.01).

To further investigate the nature of the large variance in aversive vocalizations for both the nose and ear region, we carried out further analyses on individual call types in the following section.

### 3.3. Aversive contexts: barks, screams, whimpers, out-group pant hoots

**Figure 5.**
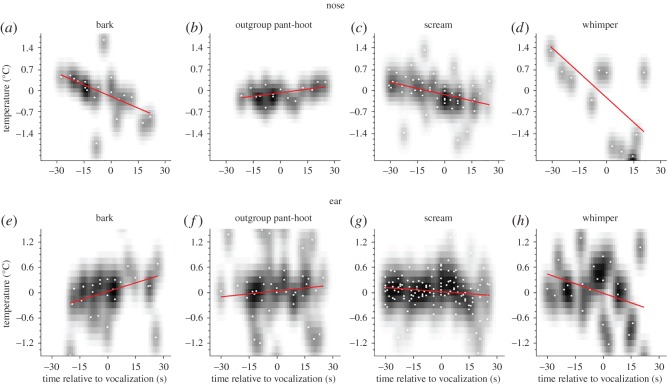
Types of aversive vocalizations. (*a*,*e*) Changes of temperature are displayed before and after the occurrence of a bark vocalization. (*b*,*f*) Changes of temperature are displayed before and after the occurrence of an outgroup pant-hoot vocalization. (*c*,*g*) Changes of temperature are displayed before and after the occurrence of a scream vocalization. (*d*,*h*) Changes of temperature are displayed before and after the occurrence of a whimper vocalization. Colour codes follow figures 3 and 4. Red lines show the Pearson linear correlation between changes in temperature and time.

‘Barks’, ‘whimpers’ and ‘screams’ were associated with significant temperature *decreases* in the nose region (barks: *r* = −0.48, *n* = 19, *p* < 0.05; screams: *r* = −0.40, *n* = 49, *p* < 0.05; whimpers: *r* = −0.60, *n* = 12, *p* < 0.05), while out-group pant hoots were associated with a significant increase (*r* = 0.46, *n* = 15, *p* < 0.05; figure 5*a*–*d*). By contrast, for the ear region, we found temperature *increases* after aggressive bark vocalizations (*r* = 0.31, *n* = 26, *p* = 0.06 (trend)), and *decreases* after passive aversive screams (*r* = −0.138, *n* = 127, *p* < 0.07 (trend)), and whimpers (*r* = −0.31, *n* = 24, *p* < 0.07 (trend); figure 5*e*,*g*,*h*), but no change after out-group pant hoots (figure 5*f*: pant hoots: *r* = 0.06, *n* = 48, *p* = 0.35).

**Figure 6.**
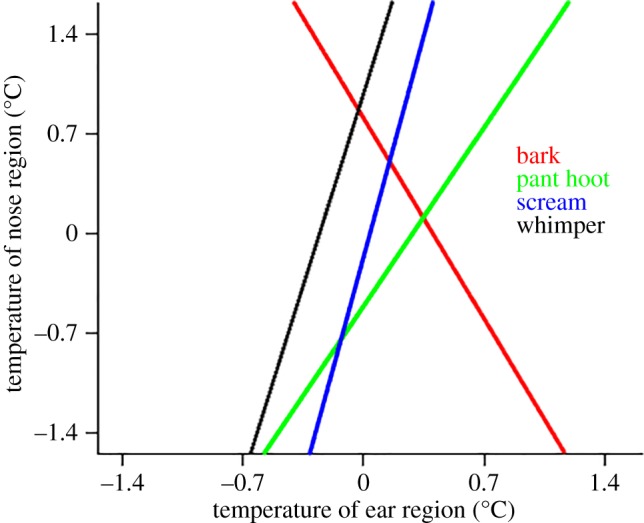
Functional characteristics of temperature changes in nose and ear regions. Shown are the polynomial functions of degree 1 (linear) of the temperature changes in the ear (*x*-axis) and the nose area (*y*-axis).

To describe directional changes in temperature across nose and ear region and across all call types, we determined the polynomial function of degree 1 (linear) of all conditions. For illustration purposes, we then plotted the function of the nose against the function of the ear for each call type. As can be seen in figure 6, ‘barks’ were the only call type that showed a converse relationship, i.e. when temperature changes were increasing in the ear, temperature changes in the nose were decreasing and vice-versa. All other vocalization types showed concurrent directional changes in temperatures.

## 4. Discussion

This study examined a physiological correlate of presumed emotional states of wild chimpanzees using infrared thermal imaging, implemented by hearing vocalizations of conspecifics. More specifically, we tested whether different types of vocalizations and the social contexts they are associated with trigger differential changes in surface body temperature. We found overall decreases in nose temperatures and increases in ear temperatures, with different vocalization relating to different thermal responses. Our premise was that, the more arousing a social event, the more the blood flow should be redirected, eventually resulting in changes in body surface temperature over the area of interest. In line with this prediction, we found larger decreases in temperature for aversive than neutral vocalizations in the nasal area. In the ear region though, we found that neutral vocalizations were associated with larger changes (increase) than aversive calls. When looking at vocalization types, our data suggest that aggressive barks, arguably the most emotionally charged intragroup vocalization, were associated with the more drastic temperature shifts.

Changes in nasal temperature are commonly reported in animals exposed to emotionally charged situations, in parallel with other physiological measures (see [20] for a review). Such changes may be a by-product of increased breathing under arousal. Alternatively, they can be related to subcutaneous vascular constriction, a phenomenon which results in reduction of blood flow in exposed body parts [16]. Regarding temperature changes in the ear, we are aware of a recent study reporting shifts in ear temperature after emotional stimulation [34]. In particular, this study found that the ear temperature of dogs decreased when their owner left, a shift also reported in macaques during alert behaviour [21]. The decrease of blood flow in the ear region was also described in rabbits submitted to an alerting stimulation [35]. These results can typically be interpreted as the consequence of vasoconstriction [16]. However, increases in ear temperature were also reported in the dogs' study, when dogs retrieved contact with the owner or experimenter, a social event associated with a positive affective reaction [34]. A similar pattern of the increase of ear temperature was present in rats exposed to fear conditioning [16]. Changes in ear temperature are a finding with considerable applied implications, especially when studying species that use gaze as a threat signal like chimpanzees. In our study, greater allocation of blood flow towards the muscles of the auricular areas may explain our pattern, increasing the performance of the auditory system, for example, by moving the pinna to enhance source localization [36]. Increased auditory input could be beneficial in socially demanding contexts, especially in a species that lives in a visually dense habitat where visual access to ongoing social events is usually highly limited.

To the best of our knowledge, our study is the first to use IRT to investigate wild chimpanzee behaviour, with the more specific aim of uncovering emotional reactions to vocalizations of conspecifics. Methodologically, our study provides relevant progress in that we were able to document significant shifts in temperature in relatively short-time windows, which is relevant for monitoring free-ranging animals. Our findings are in line with a previous study that reported thermal changes with a 10–20 s delay in captive macaques exposed to video clips of aggressive social interactions [23]. In other studies, body surface temperature changes were documented over much longer time periods, while temperature changes were higher (Δ1.5°C [24]) in comparison to our findings (Δ0.5°C—mean absolute change in skin temperature). Future research will have to focus on the factors that influence the delay and amplitude of temperature shifts, including the type of social stimulation and the presence of relevant social partners.

We see a number of limitations to our research. First, highly relevant social events (e.g. a screaming mother running to rescue her offspring in response to aggression) are absent from our dataset. This was largely because highly relevant social events, such as physical aggression, are usually associated with considerable body movements, which makes IRT monitoring very challenging. This is of course problematic because such events correspond to the most interesting and arousing social situations from the perspective of the animals.

Second, the number of significant social events (vocalizations, displays, aggressions, etc.) naturally occurring in wild chimpanzees is so rich that it is difficult to assign thermal changes to a single social element. This is particularly clear for agonistic interactions where calls (such as barks and screams) start being produced after the initiation of aggression. Figure 5*a*,*e* is particularly illustrative of this as they suggest that the decrease of the nasal temperature and the increase over the ear area may well start before a bark is produced. Yet, vocalizations may act as key behaviours in such contexts as they signal to others the severity of aggression. Future studies with wild animals should collect a sufficiently large number of series to be able to select the most detailed ones and to better single out the contribution of calling behaviour to thermal changes in hearers. Another viable methodological strategy would be to use playbacks so that a significant social event cannot be foreseen before a vocalization is being produced. For now, we may not therefore be in position to establish that our shifts in temperature reflect *causation* by the calls themselves.

Third, we did not collect social data (such as social network indices, dominance, aggression, females' reproductive state) that could have helped interpret in more detail the emotional significance of each vocal event for the subjects. In addition, we only had limited information on the calling chimpanzees, which is crucial to precisely evaluate the arousing nature of the vocalizations. Future studies should collect those data in a more systematic manner.

Finally, our IRT data have not been grounded in other physiological variables normally linked with emotional states, such as cardiac activity, galvanic skin response or cortisol changes. In wild animals, including chimpanzees, hormonal changes in oxytocin and cortisol linked to social events have been determined successfully from urine [37–40]. If no changes in salivary cortisol could be found in a study with captive chimpanzees exposed to videos of agonistic interactions in conspecifics [24], future investigations should consistently include hormonal measurements.

Altogether, our study encourages the use of IRT with wild chimpanzees. It provides a basis for a more systematic analysis of how vocalizations and the social events they are associated with may modulate emotional responses in animals, with the associated changes in skin temperature that can now be reliably measured.

## Data accessibility

All relevant data are available from the Dryad Digital Repository: http://dx.doi.org/10.5061/dryad.dt7mr [41].
